# Health care agreements as a tool for coordinating health and social services

**DOI:** 10.5334/ijic.1452

**Published:** 2014-12-15

**Authors:** Andreas Rudkjøbing, Martin Strandberg-Larsen, Karsten Vrangbaek, John Sahl Andersen, Allan Krasnik

**Affiliations:** Center for Healthy Aging, Section for Health Services Research, Department of Public Health, University of Copenhagen, Denmark; Section for Health Services, Department of Public Health, University of Copenhagen, Denmark; Section for Health Services Research, Department of Public Health, University of Copenhagen, Denmark; Section for General Practice, Department of Public Health, University of Copenhagen, Denmark; Center for Healthy Aging, Section for Health Services Research, Department of Public Health, University of Copenhagen, Denmark

**Keywords:** integrated care, coordinated care, health planning, National Health System, Denmark

## Abstract

**Introduction:**

In 2007, a substantial reform changed the administrative boundaries of the Danish health care system and introduced health care agreements to be signed between municipal and regional authorities. To assess the health care agreements as a tool for coordinating health and social services, a survey was conducted before (2005–2006) and after the reform (2011).

**Theory and methods:**

The study was designed on the basis of a modified version of Alter and Hage's framework for conceptualising coordination. Both surveys addressed all municipal level units (*n* = 271/98) and a random sample of general practitioners (*n* = 700/853).

**Results:**

The health care agreements were considered more useful for coordinating care than the previous health plans. The power relationship between the regional and municipal authorities in drawing up the agreements was described as more equal. Familiarity with the agreements among general practitioners was higher, as was the perceived influence of the health care agreements on their work.

**Discussion:**

Health care agreements with specific content and with regular follow-up and systematic mechanisms for organising feedback between collaborative partners exemplify a useful tool for the coordination of health and social services.

**Conclusion:**

There are substantial improvements with the new health agreements in terms of formalising a better coordination of the health care system.

## Introduction

Integration of services within and across sectors and over time is a major concern for policy-makers in most western countries [1, 2]. The epidemiological transition, changes in socio-demography and the consequential increase in the number of patients living with chronic disease are changing the demands for health care [3, 4]. Patients with chronic diseases drive the need for comprehensive, coordinated, long-term and multifaceted prevention, treatment and rehabilitation services [4]. When these are poorly coordinated, the right services at the right time fail to be delivered or may be duplicated [5], and risk increases with consequences for quality [6], safety [6] and patient experience [7, 8]. Similarly, the potential for savings or cost-effectiveness diminishes [5, 6, 9, 10]. Denmark is divided into three political and administrative levels: the state, five regions and 98 municipalities. This is also reflected in the health care system which is a tax-financed Beveridge-type system, where the state is responsible for overall financing and regulation. Most secondary and tertiary care takes place in hospitals owned and operated by the regions. General practitioners, who act as gatekeepers to specialist care, are private (self-employed) and financed by the regions through a mixture of capitation and fee for service [11]. Prevention, rehabilitation (outside hospitals) and home care are among the main responsibilities of the municipalities and the same is the case for social care which, however, is not part of the health services. Health care at the hospitals and at the general practitioner is free at the point of service.

The Danish health system has, with its regional centre of gravity (see Box 1), the potential to provide integrated care, but due to cultural, structural and managerial challenges [12], the effort falls short and there are concerns regarding the coordination of service provision within and across hospital care, primary care and municipal care services [1, 13, 14]. This is despite a stated goal of coherent services in the first paragraph of the Danish health act, a long tradition of joint working between health and social care [1] and a major reform of the public sector with the aim of improving coordination of care [1, 12, 15, 16].

The present study aims to assess health care agreements as a tool for coordinating health and social services. Health care agreements were introduced as part of large public sector reform in 2007. Our study is a follow-up to one from 2007 that assessed the previous health plans that were replaced by the new agreements [17].

### A major public sector reform

Historically, health care management and service provision were largely decentralised [1, 18, 19]. A major structural reform in 2007 was the culmination of a series of interventions over the decade that attempted to strengthen coordination and centralise control [20, 21]. The reform merged 15 units (all counties plus Copenhagen, Frederiksberg and the Regional Municipality of Bornholm) into 5 regions and the number of municipalities from 271 to 98.

Another element of the reform was the introduction of health care agreements as a tool for improving the coordination of health and social care. The agreements are negotiated every four years at the beginning of an election cycle between the municipality and the corresponding region [22]. Health care agreements describe patient pathways across the health and social care services (see Box 2) and are a form of virtual vertical integration [23] or a ‘contractual’ form of organisational integration [24, 25]. They cover six specific policy areas with questions that must be addressed and with the option of adding others [22].

The six mandatory areas include:

- Hospitalisation and discharge processes (see Box 2)
- Rehabilitation
- Devices and aids
- Prevention and health promotion, including patient-focused prevention
- Mental health
- Follow-up on adverse events

A national monitoring system reports on indicators related to the health care agreements such as waiting time for rehabilitation, avoidable readmissions and discharge processes [26].

The health care agreements are a further development of the health plans, which were county-based documents including a description of the health status of the population, available services and the nature and extent of collaboration with the respective municipalities and other counties. The health plans were drafted on the basis of meetings, seminars and joint committee work and were sent to the Health and Medicines Authority for comments [17]. The health plans were assessed in a study before the 2007 reform [17] and showed that they were not effective as a coordination tool and that the counties primarily made the decisions on the content of the health plans. The study also found limited awareness and influence of the health plans among general practitioners. With the new health care agreements followed a number of important changes that possibly changed the awareness and perception of their usefulness and balance of power in the negotiation, although the actors remained the same. The changes include more stringent requirements for content and central approval, monitoring and feedback which presumably lead to the health care agreements being a more highly prioritised topic on the political agenda.

The research evidence of joint working suggests that significant benefits can be achieved [27, 28], but many contributions also point to significant challenges (15). The evidence on specific planning tools remains limited [17]. We conducted surveys in municipalities and general practice before and after the reform with the aim of providing a quantitative research-based assessment of health care agreements as a tool for improving care coordination and as a comparison with the assessment of the health plans from before the reform.

## Theory and methods

### Theoretical background

The theoretical background for this study is a modified version of Alter and Hage's framework for conceptualising coordination [28], which can be seen as part of the broader set of inter-organisational network theory/soft system theory [29]. The health system can thus be seen as a complex structure of organisations or sub-systems interacting in specific patterns and governed by a combination of hard and soft regulation combining incentives, sanctions and normative pressure [30]. To cope with the increasing complexity and need for resource efficiency, health care systems differentiate and specialise their units. As a response to this and the demands for integrated services, better coordination is needed.

According to Alter and Hage, coordination in practice, at the micro level, is dependent on the local stakeholders’ willingness to cooperate, thus making the perceptions of these stakeholders highly important in the process of assessing the preconditions for coordination. Alter and Hage describe two different levels that must be coordinated: administrative and operational. The administrative level is defined as interagency coordination that happens at the level of senior management and administrator and the operational level as the coordination activity that happens among frontline staff. Coordination within and between more complex systems requires a high degree of feedback [28]. Governance mechanisms to improve coordination must facilitate such feedback and structure collaborative practices to overcome potential barriers. Appropriate coordination governance may thus contribute to better system performance.

Introducing larger municipalities in Denmark should, in principle, strengthen the administrative capacity to apply coordination governance mechanisms within the municipalities and negotiate and implement health agreements with the regional health systems. However, there is a risk of the asymmetric power relation persisting, since in-hospital planning is traditionally given more weight than out-patient services and preventive efforts.

From a theoretical perspective, the health care agreements represent substantial improvements in terms of formalising a better coordinated care system compared to the previous health plans [17]. The health care agreements are anchored in regional consultative committees with representatives from the region, the municipalities and general practitioners. As such, they function simultaneously as a political agreement that provide the framework for the joint health planning between the administrative levels and as structures for practical cooperation between the providers. They provide central oversight as well as a feedback mechanism as they must be submitted to health and medicines for approval based on whether they meet requirements formulated in ministerial guidance notes [22].

The health care agreements seem theoretically appropriate for governing the service-providing network between the region and the municipality. The legislative set-up, the specific guidelines, the central oversight and monitoring indicate serious central attention, while keeping the task division and the more operational decisions to the service providers defined as the regional and municipal health authorities, however, with a special focus on the political and administrative level and less on the operational level of clinical health professionals in hospitals and primary care.

Rhodes and other scholars have described the shift from government to governance and from hierarchy to networks and the consequential change in the use of policy instruments from hard to soft over the past decades [31–33]. Hard regulation is increasingly difficult to apply since the state relies on the cooperation of a plethora of local, regional and non-government actors in a process where the application of coercion might undermine the actors’ willingness to cooperate [33, 34]. In this context, this policy is an interesting and potentially effective fusion of hierarchical steering and cooperative, network-based steering in a multilevel governance setting. Compared to the previous health plans, we expect that the content requirements of the health care agreements defined and approved by the central government and the stronger emphasis on the municipal responsibilities after the reform could potentially ensure a more equal power relation and balanced weighting of regional and municipal policy domains.

### The survey questionnaire

The questionnaire items of the baseline survey were based on a literature review and part of a multilevel survey to provide empirical data about the structural reform. The baseline survey was conducted in 2005–2006 and the follow-up in January–April 2011, leaving time for changes and effects of the reform to develop [35, 36].

The baseline study included municipalities, counties and general practitioners using similar items for all three levels to enable comparisons of the perception of the health care agreements between the main actors. After the reform, however, the 15 units originally surveyed at the regional level (all counties plus Copenhagen, Frederiksberg and the Regional Municipality of Bornholm) were merged to five regions. Even though we did send surveys to the administrative managers at the regional level, only one manager completed the questionnaire. We therefore only report survey data for municipalities and general practitioners in the following (Table 1).

The wording of the questionnaire items was formulated after a two-step testing procedure. The first was a peer review process among health service researchers; the second was a pilot study among representatives from each respondent group. This was done to improve face and content validity. To allow for comparisons between the baseline and follow-up surveys, we maintained the same wording for a number of items, except that ‘health plans’ was substituted with ‘health care agreements’.

Contact details of general practitioners were obtained from the general practitioners’ organisation register. For the other two groups of respondents, no random selection was necessary since all were invited to participate in the survey. The municipal directors of social and health affairs were identified through the Association of Directors of Social and Health Affairs and the information was confirmed by telephone when necessary.

The baseline survey was conducted as a postal survey and double keyed-in using EpiData (www.epidata.dk) and the follow-up as a web-based survey using Surveyxact©. The postal and web-based surveys were designed to allow the respondents to maintain anonymity, and two postal/e-mail reminders were sent to increase the respondent rate.

## Results

### Analysis of quantitative data

The baseline response rate for directors of social and health affairs was 62.4% (*n* = 169) and for general practitioners 63.1% (*n* = 442). The follow-up response rate for directors of social and health affairs was 56.1% (*n* = 55) and for general practitioners 50.1% (*n* = 378).

Missing data on the relevant items for this paper were excluded, leaving 163 directors of social and health affairs and 429 general practitioners for the analysis for the baseline survey and 55 directors of social and health affairs and 378 general practitioners for the follow-up survey. To test for non-response bias we tested, in both baseline and follow-up, whether the survey groups were representative. For the municipalities, we did not have the information to perform non-response analysis so the analysis was done for the general practitioners only. The distribution of gender and practice type was known for general practitioners on a national level [37]. That allowed us to compare the distribution of characteristics with the data reported by responders to the general practitioner survey. We used a binominal test of proportions. The responders were representative regarding gender on a 5% significance level in both the baseline and follow-up surveys [37]. There were a significantly higher number of partnership practices among the responders in both baseline and follow-up - 69.5% compared to the national distribution 63% at baseline and 70% compared to a national distribution of 65% at follow-up. We tested for skewness in the survey participation of the general practitioners across the different regions and based on organisational differences (group practice versus single practice) and found no significant differences.

The perceived influence of health plans and health care agreements in municipalities and in general practice was analysed by descriptive statistics. Fisher's exact test was used to assess the baseline and follow-up difference in perceptions between the respondents in the municipalities and among general practitioners.

### Data presentation

The health care agreements as a tool for strengthening the coordination, quality and preventive services between county/region and the municipality were more positively assessed than the health plans at baseline (Table 2). Twenty-five per cent of the directors of health and social affairs at follow-up considered that the health care agreements were “to a high degree” helping to secure coordination and quality versus 5% at baseline. Sixty-four percent at follow-up versus 44% at baseline answered “to some degree” and 9% versus 44% “to a lesser degree”.

At follow-up we can observe a more balanced perception of the power-balance between the parties (Table 3). At baseline in assessing the relative strength of the parties, 87% of the directors of health and social affairs answered that decisions on the health plans were predominantly made by the regional authorities; after the reform only 47% of respondents reported that this was the case. Twelve percent at baseline versus 51% at follow-up reported that the two actors had an equal strength in the process, whereas 2% versus 0% said that the municipalities were stronger.

At the functional level of health care, among the general practitioners, a large proportion (16%) were not familiar with the health care agreements at follow-up, despite a clear drop from 27% at baseline (Table 4). More general practioners at follow-up reported that the health care agreements had an influence on their work - 47% (combined “to a high degree” and “to some degree”), up from 29% at baseline.

## Discussion

Health care agreements are an interesting case for integrating health and social care because they represent a break with the traditional sector-based management and leave a network organisation, the health coordinating committees with administrative and professional actors from both regions and municipalities, in charge of overseeing development and monitoring. From a theoretical perspective, this is an interesting fusion of hierarchical steering and cooperative, network-based steering in a multilevel governance setting. However if implemented incorrectly, the new set-up can result in a “re-disorganisation” of established collaborative relationships, with potentially negative effects for patient pathways and coordination of services [38].

From a practical perspective, there are substantial improvements with the health care agreements in terms of formalising a better coordination of the health care system. Planning of the health care agreements is treated, to a high degree, as a continuous learning and adaptation process, with continuous follow-up on the agreements in the regional consultative committees. This provides a facilitating platform allowing for continuous use of administrative coordination methods with a high degree of feedback, which is theoretically more appropriate for coordinating a highly complex inter-organisational network such as the Danish health system [39].

Our study found that the health care agreements were considered more useful than the previous health plans. In a qualitative study by Vrangbaek [40], the municipalities reported that they felt that not only the health care agreements were a useful tool, but also they were highly resource-intensive and bureaucratic. Nielsen's qualitative study of the dynamics between Region Zealand and 17 corresponding municipalities found that the regions thought that the process was time-consuming and costly and wanted standardisation as much as possible across the 17 different health care agreements. On the other side, the municipalities were working to tailor the health care agreements to their specific municipal structure or practice [41].

The fact that the municipalities are larger and that the content of the agreements is centrally defined might have made the power relationship less asymmetric. Some asymmetry is to be expected but could still be an important barrier to joint working and whole-system planning if the municipalities have their priorities overruled. That would limit the usefulness in terms of coordination from the perspective of the municipalities. Vrangbaek's study also found that the municipalities considered the relative strength to be unequal and that they saw a need for inter-municipal coordination to strengthen their negotiating position [40]. The regions, on the other hand, felt that the relative strength between them and the municipalities was pretty equal [40]. A qualitative study of the members of the health coordinating committee [42] found that an ‘equal partnership’ between the region and the municipalities was crucial in the process of coordinating health and social care and that the rhetoric of an equal partnership was actively promoted in order to change the perception of a somewhat conflict-ridden [42] and unequal relationship [17, 43].

At the functional level, the general practitioners were more familiar with the health care agreements than the previous health plans and reported that they have a more significant influence on their work. Still, less than half of them experience that the health care agreements influence their work ‘to some’ or ‘to a high degree’. Vrangbaek found that the general practitioners lacked knowledge about the health care agreements [40] but found them useful even if they were sceptical about standardisation and bureaucracy. General practitioners are key figures for the success of the health care agreements and it is a well-known challenge to include primary care in integrated care interventions [44–46]. In spite of this, the parties to the agreement are solely the regional and municipal authorities. Representatives of the general practitioners participate as observers in the negotiation and follow-up of the agreements, but the general practitioners are not formally bound by them [47–49].

There are risks that coordination efforts at the administrative level do not fundamentally alter how physicians and other frontline staff collaborate with professionals [23–25]. Although Nielsen's and Vrangbaek's studies [40, 41] concluded that the health care agreements and the health consultative committees were successful in engaging the regions and municipalities in a dialogue on how to solve their shared challenges of integrating services, it is the work that comes after the signing of the health care agreements, the articulation, adjustments and adaption of the health care agreements in practice that produces coordination. A study from 2010 [50] of frontline staff at the hospital concluded that there was widespread knowledge about the agreements, but that they were not fully implemented. The study showed that it remained difficult to get the message across to all members of staff and that the agreements did not solve all problems regarding admission and discharges, although they did create a forum for dialogue and common understanding.

A response rate of between 50% and 60% means that the possible impact of selection bias must be considered because of the self-selection element inherent in this kind of survey [17]. The nature and effect of bias included in the study is difficult to predict and might vary and affect the research in opposite ways. The items reported here are parts of a larger survey, with multiple themes making the potential selection bias specifically for the reported items less likely. For the municipalities, we did not measure background variables and therefore could not perform a non-response analysis, but we consider that the most likely bias would be an underestimation of the true coordination problems, due to a strategic wish of the stakeholders to present themselves in a positive light. Responses from the administrative level are also likely to be more positively evaluated than from the employees at the frontline [40, 41] Assuming that the potential bias is stable between the two surveys, there is a clear trend and a significant change in the items reported.

## Conclusion

Our study shows that the health care agreements are considered a useful tool for strengthening coordination between the regions and municipalities and are more positively assessed as a tool for service coordination than the previous health plans, but that challenges still persist. The improved performance of the health care agreements in comparison with previous health plans confirms our theoretical prediction that a coordination method with a high degree of feedback is suitable when coordinating more complex system where standardisation is not an option. As such, the health care agreements represent an example of “soft regulation”, which may be particularly well-suited to regulate complex interactions between decentralised public authorities to promote coordination and development, while at the same time maintaining a common framework to secure some degree of uniformity and allow central level evaluation.

## Figures and Tables

**Box 1.**
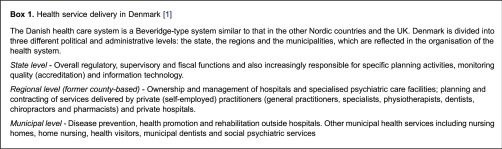
Health service delivery in Denmark

**Box 2.**
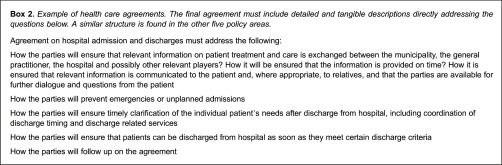
Example of health care agreements. The final agreement must include detailed and tangible descriptions directly addressing the questions below. A similar structure is found in the other five policy areas.

**Table 1.**
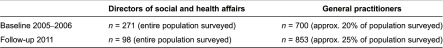
Overview of surveyed population of all directors of social and health affairs and a sample of general practitioners in Denmark

**Table 2.**
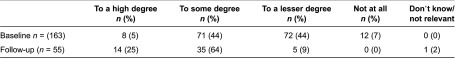
Assessment of the health plans/health care agreements as a tool for strengthening coordination, quality and preventive services between the county and the municipalities: “*To what degree do you consider the joint health plans/health care agreements, developed every four years, fulfil their objective of strengthening the coordination, quality and preventive services between the county and municipalities?*” *(Responses from municipal directors of social and health affairs)*

*p* values for Fisher's exact test = <0.0001.

**Table 3.**
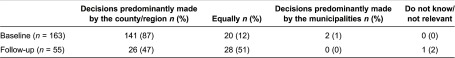
Assessment of the relative strength between the county/region and the municipalities in developing health plans/health care agreements: “*How would you describe the relative strength between the county/region and the municipalities in the development of the health plans/health care agreements?*”

*p* values for Fisher's exact test = <0.0001.

**Table 4.**
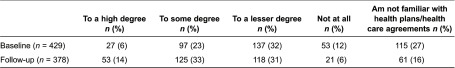
Assessment of the influence of the health plans on work as a general practitioner: “*To what degree have you experienced that the joint health plans/health care agreements between the county/region and the municipalities influence your work?*”

*p* values for Fisher's exact test = <0.0001.
